# Supplementary material for: CircRNAome of Childhood Acute Lymphoblastic Leukemia: Deciphering Subtype-Specific Expression Profiles and Involvement in TCF3::PBX1 ALL
Source: Int J Mol Sci. 2024 Jan 25;25(3):1477. doi: 10.3390/ijms25031477 (PMC10855129; doi:10.3390/ijms25031477)
Supplement: Supplementary file 1 [file ijms-25-01477-s001.zip › SuplementraryFigures_circRNAs_B_ALL_20240115.pdf]

A) B-cell Controls

Input Sample	earlyB	proB	preB	ImmatureB	P-value	Correlation
cord_27844	0	0.128	0	0.872	0.000	0.867
cord_27846	0	0.106	0	0.894	0.000	0.884
cord_27849	0	0.054	0	0.946	0.000	0.909
ALLMIRC007-Bcells	0	0.004	0.116	0.880	0.000	0.954
ALLMIRC011-Bcells	0	0.022	0	0.978	0.000	0.956
ALLMIRC012-Bcells	0	0.026	0	0.974	0.000	0.972
ALLMIRC014-Bcells	0	0.01	0	0.990	0.000	0.983

B) Leukemia samples

Input Sample	earlyB	proB	preB	ImmatureB	P-value	Correlation
DUX4 D1	0.024	0.896	0	0.080	0.000	0.746
DUX4 D2	0	0.949	0.051	0.000	0.000	0.789
DUX4 D3	0.056	0.848	0	0.096	0.000	0.914
ETV6::RUNX1 D1	0.003	0.947	0	0.049	0.000	0.665
ETV6::RUNX1 D2	0	0.957	0	0.043	0.000	0.734
ETV6::RUNX1 D3	0.099	0.806	0.095	0.000	0.000	0.858
HHD D1	0.147	0.63	0.13	0.093	0.000	0.911
HHD D2	0.037	0.956	0	0.006	0.000	0.927
HHD D3	0	0.94	0	0.060	0.000	0.945
MLL D1	0.092	0.889	0	0.019	0.000	0.884
MLL D2	0.043	0.921	0	0.036	0.010	0.526
MLL D3	0.077	0.883	0	0.040	0.000	0.764
TCF3::PBX1 D1	0.012	0.881	0.091	0.016	0.000	0.886
TCF3::PBX1 D2	0.016	0.92	0.064	0.000	0.000	0.777
TCF3::PBX1 D3	0	0.981	0	0.019	0.000	0.872
Phi-like D1	0.01	0.882	0.077	0.032	0.000	0.957
Phi-like D2	0.031	0.898	0	0.070	0.000	0.856
Phi-like D3	0.104	0.845	0	0.051	0.000	0.827

Figure S1: Predominant hematopoietic cell signal within each bulk transcriptome compared to early B (CD34+/CD19-/IgM-), pro-B (CD34-/CD19+/IgM-), preB (CD34+/CD19+/IgM-) and immature cells (CD34-/CD19+/IgM+) in **A**) controls and **B**) leukemia samples representing six subtypes of B-ALL from the discovery cohort.

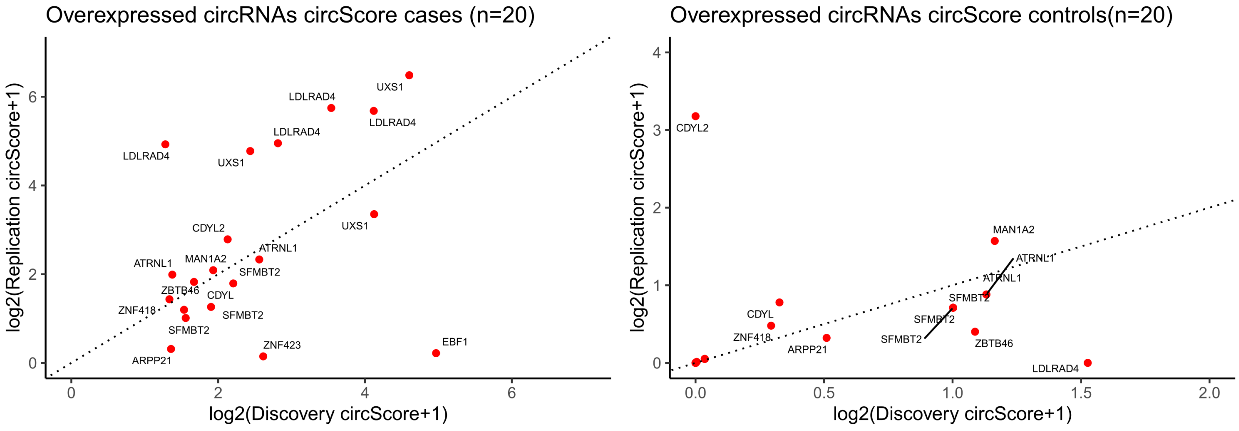

Figure S2: CIRCscores of the top scoring circRNAs (n=20) that are overexpressed in subtypes. Right: Mean CIRCscores of cases in the discovery and replication cohorts. Left: Mean CIRCscores of controls in the discovery and replication.

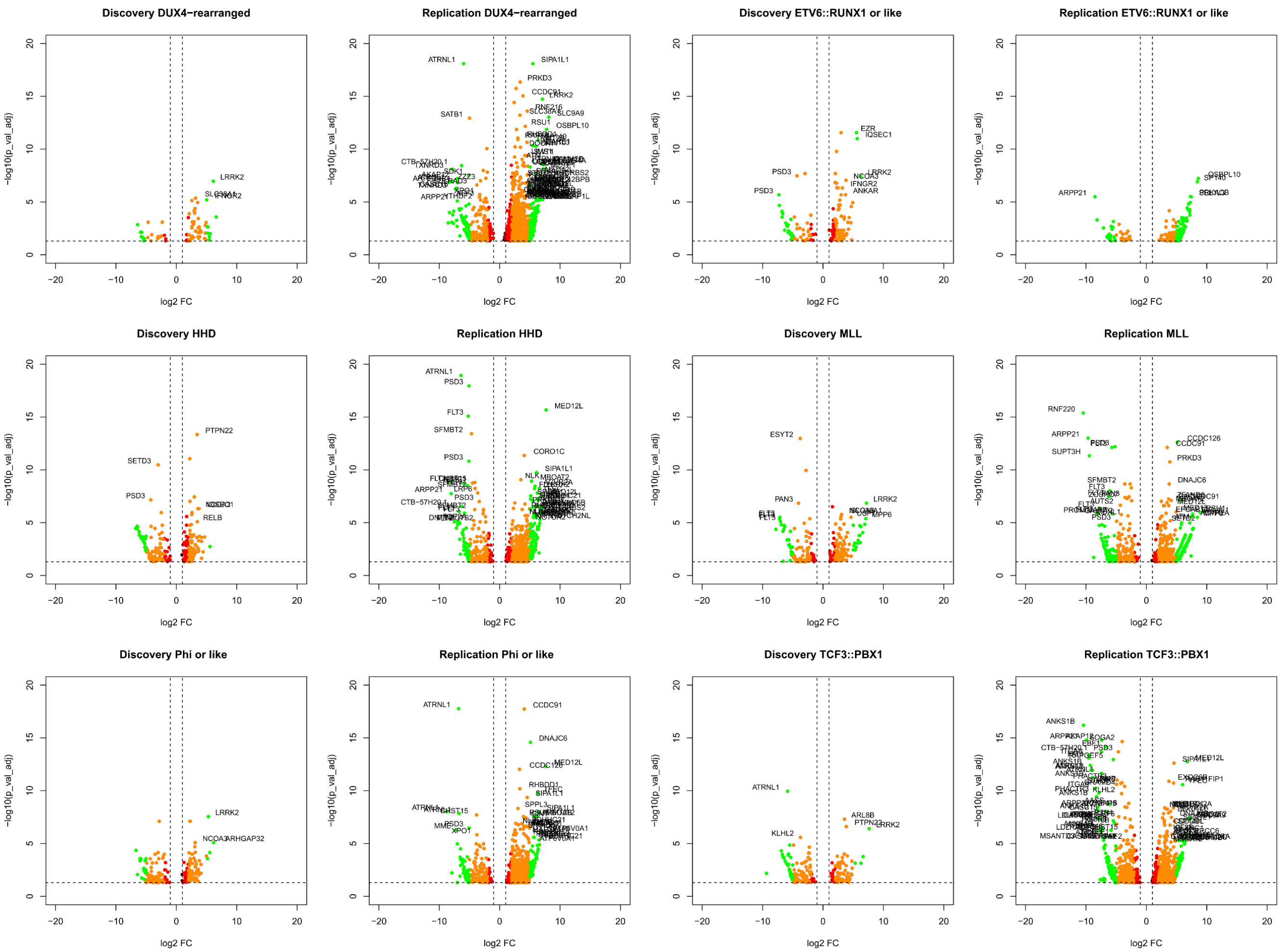

Figure S3: Volcano plots of the most deregulated circRNAs in each cohort by subtypes.

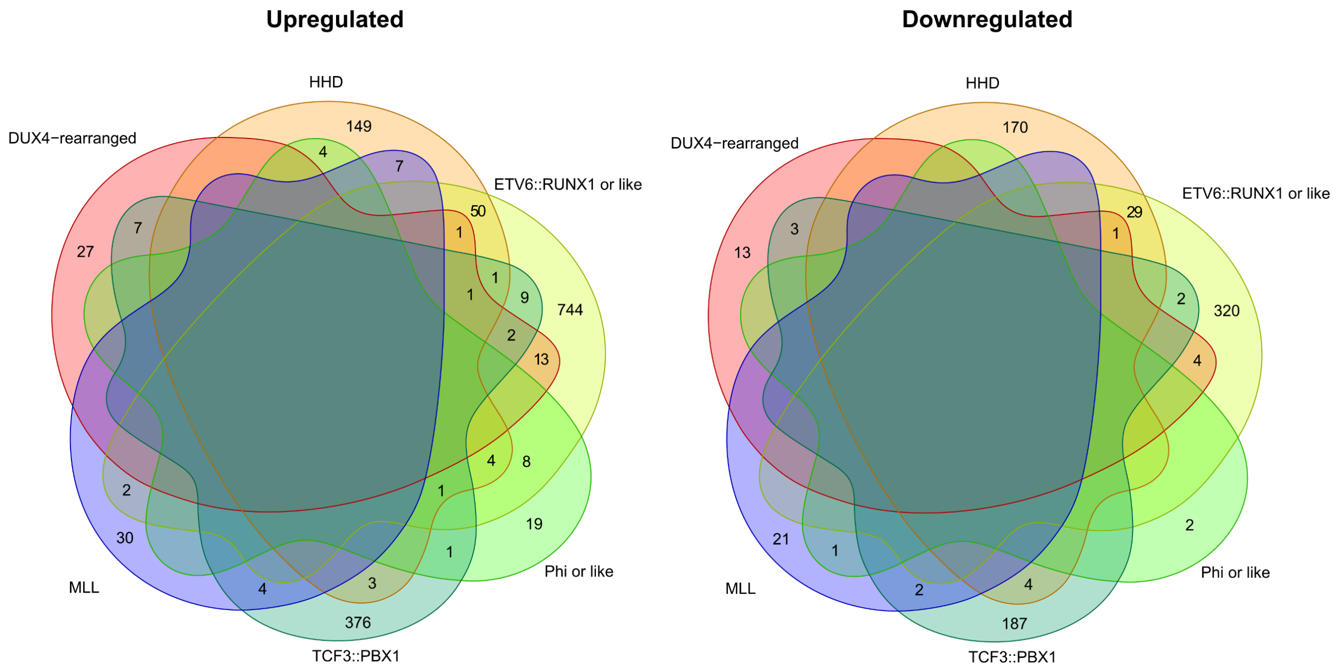

Figure S4: Venn diagram of overexpressed and downregulated circRNAs for each subtype compared to the rest of the subtypes in the discovery cohort

Discovery

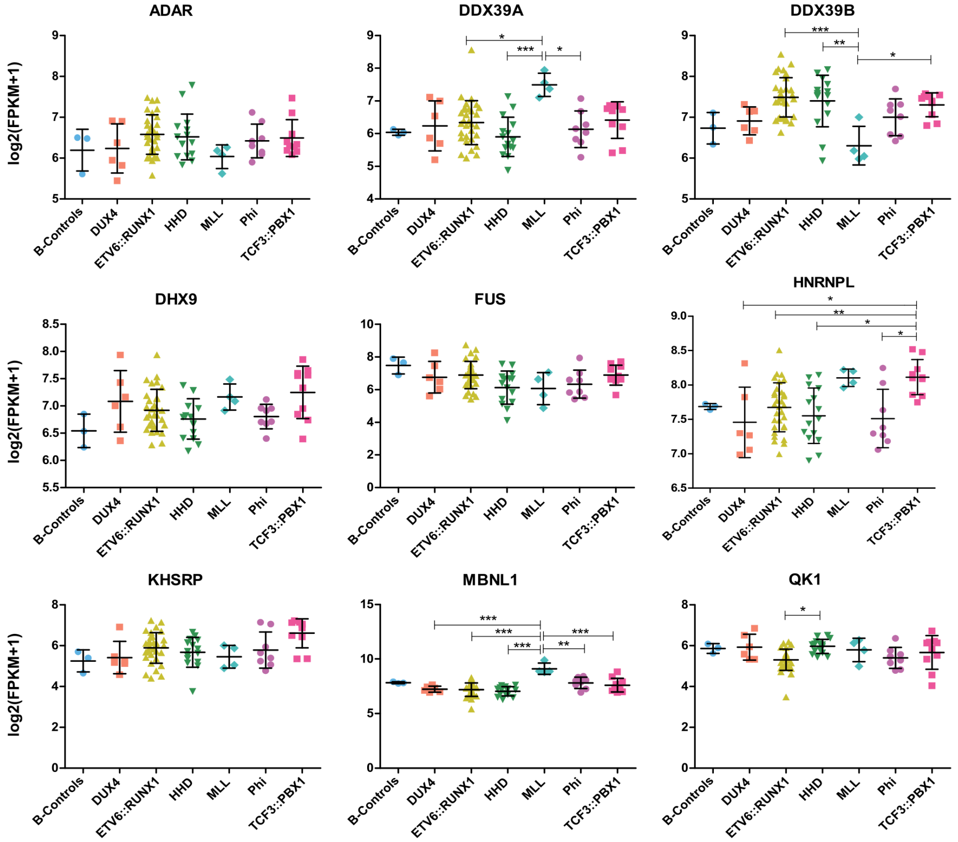

Replication

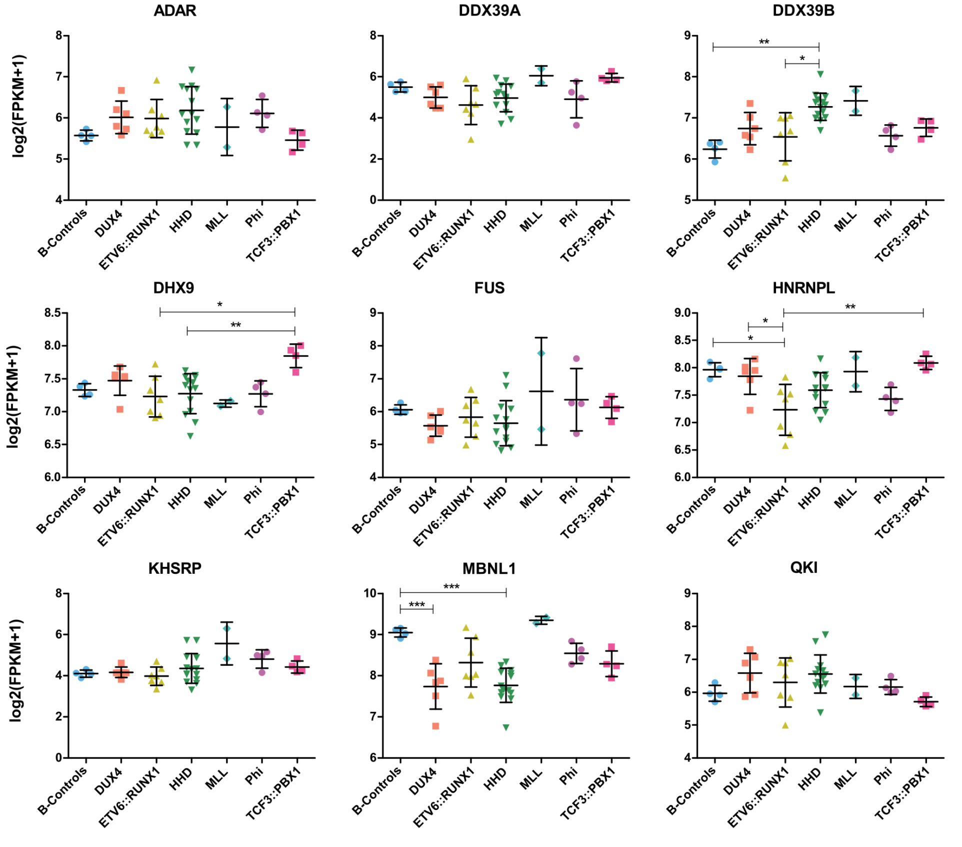

Figure S5: Biogenesis of circRNAs. FPKM values for 9 additional genes involved in circRNAs biogenesis (complement to Figure 3A). Values from the discovery and replication cohorts were extracted from transcriptome data and log transformed. Only significant p-values (<0.05) are indicated (Anova test, corrected for multiple testing with Bonferroni).

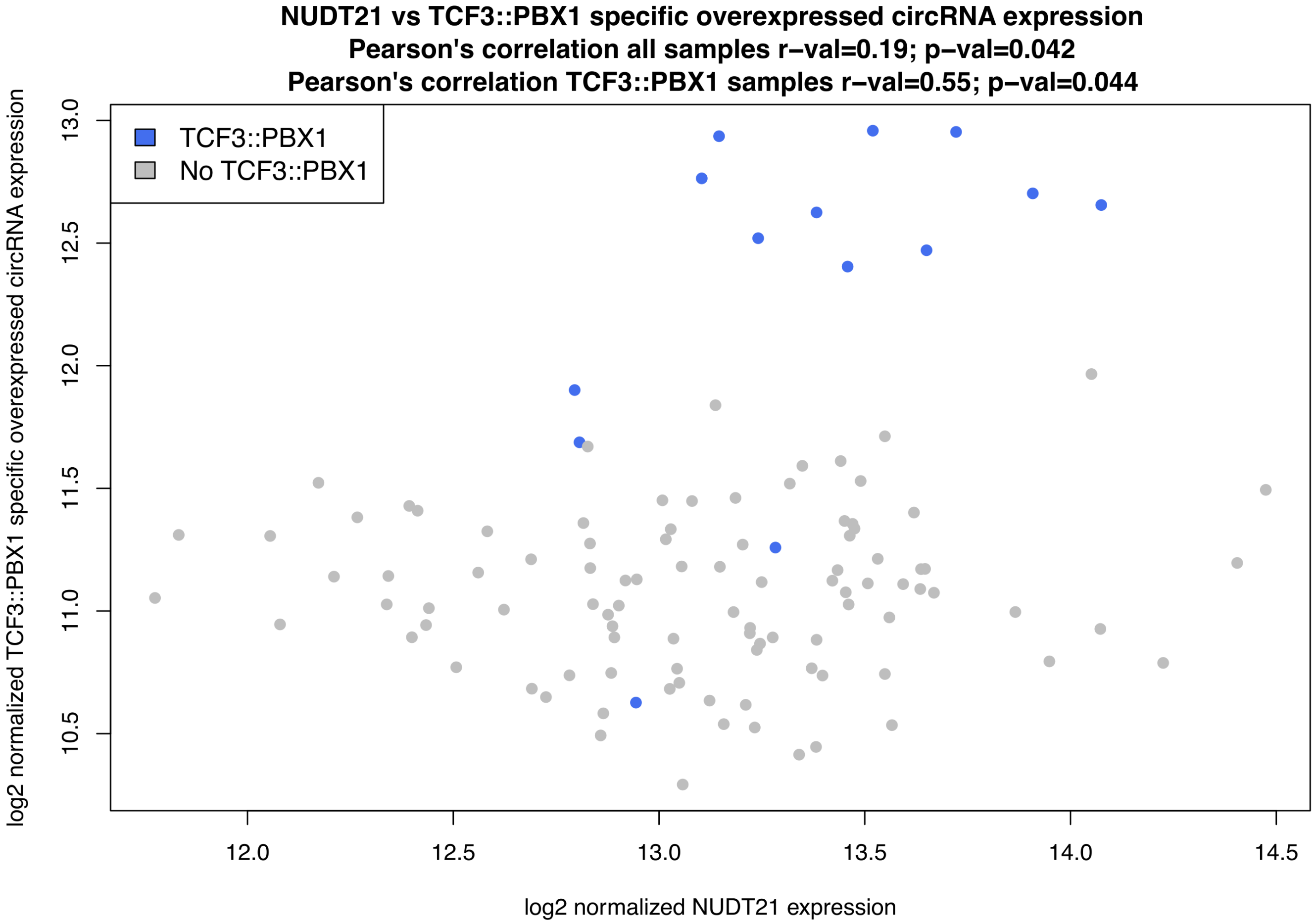

Figure S6: Correlation analysis showing global expression of upregulated candidates identified in TCF3::PBX1 patients (82 circRNAs) and NUDT21 expression.

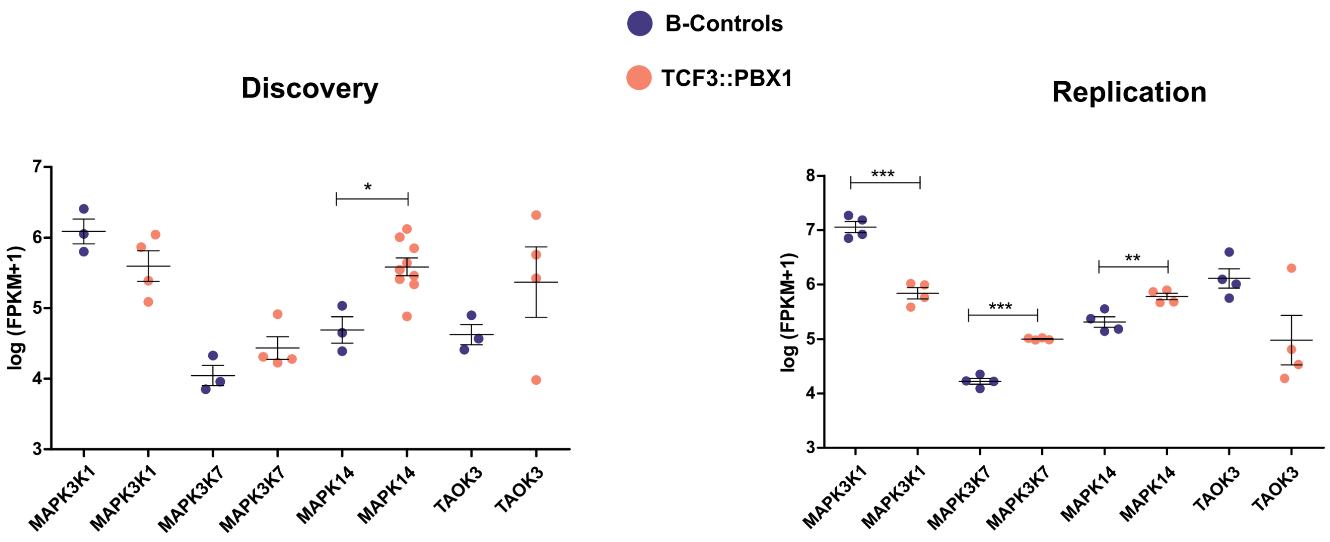

Figure S7: Expression analysis of p38 MAPK signaling pathway genes. FPKM values of *MAKP14* (p38), *TAOK3*, *MAP3K1* and *MAP3K7* in the discovery and validation cohorts were extracted from the transcriptome data and log transformed. Only significant p-values (<0.05) are indicated (t-test).
